# Notes

**Published:** 1896-11

**Authors:** 


An interesting little book has been issued by the State Board of Veterinary Medical Examiners of Maryland, containing a copy of their law, list of registrations under the Act and laws creating a State Veterinary Sanitary Board, and applicable to the control and suppression of infectious and contagious diseases among live-stock. It is neatly printed and will prove a ready reference book. Other State Boards would do well to issue similar records in book-form.

---

Dr. Benjamin Lee, Secretary of the Pennsylvania State Board of Health, will address the Keystone Veterinary Medical Association on Tuesday evening, November 10, 1896, at 9 P.M., at the northwest corner of Broad and Filbert Streets, third floor. Topic: "Subjects for future Legislation of Mutual Interest to the Medical and Veterinary Professions." The officers of the Society extend a cordial invitation to all who are interested in this work, and to the veterinarians of Philadelphia and vicinity especially.

---

The following bulletins of great importance to the veterinary profession have been received recently:

From the United States Department of Agriculture: "Vivisection in the District of Columbia;" No. 10, "Cornstalk Disease and Rabies in Cattle;" No. 25, "Dairy Bacteriology;" No. 29, "Souring of Milk and Other Changes in Milk-products;" No. 42 (Farmer's), "Facts about Milk."

From Alabama Agricultural Experiment Station: No. 72, "A Study of Skin-tumors of Horses and Mules in Alabama."

From Hatch Experiment Station: No. 41, "On the Use of Tuberculin."

From the State Board of Health of Massachusetts: "On the Prevention of Tuberculosis."

From Agricultural Station, Purdue University: "Intestinal Parasites of Sheep."

From Louisiana State Experiment Station: Bulletin No. 43 (second series).

